# Effect of Alcohol Structure on the Optimum Condition for Novozym 435-Catalyzed Synthesis of Adipate Esters

**DOI:** 10.4061/2011/162987

**Published:** 2011-12-27

**Authors:** Mohd Basyaruddin Abdul Rahman, Naz Chaibakhsh, Mahiran Basri

**Affiliations:** ^1^Department of Chemistry, Faculty of Science, Universiti Putra Malaysia, 43400 Serdang, Malaysia; ^2^Structural Biology Research Center, Malaysia Genome Institute, MTDC-UKM, Smart Technology Centre, UKM Bangi, 43600 Bangi, Selangor, Malaysia

## Abstract

Immobilized *Candida antarctica* lipase B, Novozym 435, was used as the biocatalyst in the esterification of adipic acid with four different isomers of butanol (n-butanol, sec-butanol, iso-butanol, and tert-butanol). Optimum conditions for the synthesis of adipate esters were obtained using response surface methodology approach with a four-factor-five-level central composite design concerning important reaction parameters which include time, temperature, substrate molar ratio, and amount of enzyme. Reactions under optimized conditions has yielded a high percentage of esterification (>96%) for n-butanol, iso-butanol, and sec-butanol, indicating that extent of esterification is independent of the alcohol structure for primary and secondary alcohols at the optimum conditions. Minimum reaction time (135 min) for achieving maximum ester yield was obtained for iso-butanol. The required time for attaining maximum yield and also the initial rates in the synthesis of di-n-butyl and di-sec-butyl adipate were nearly the same. Immobilized *Candida antarctica* lipase B was also capable of esterifying tert-butanol with a maximum yield of 39.1%. The enzyme is highly efficient biocatalyst for the synthesis of adipate esters by offering a simple production process and a high esterification yield.

## 1. Introduction

Natural and synthetic esters are essential materials in chemical industry. They have been most commonly applied in manufacturing of lubricating oils, solvents, plasticizers, paints, food, pharmaceuticals, cosmetics, and liquid fuels [1]. Among the esters, dicarboxylic acid esters are of particular interest due to their excellent properties such as low volatility, high flash point, good thermal stability, and low toxicity [2]. Up to now, the processes of esterification in industry are still catalyzed by chemical catalysts mainly sulfuric acid. However, difficulties in the recovery of catalyst, high energy consumption, corrosion of equipments, and the necessity for treatment of wastes are disadvantages in the chemically catalyzed synthesis [3]. In recent years, the use of enzymes for carrying out esterification reactions has been extensively studied. In comparison with chemical catalysts, enzymes show higher specificity and selectivity, they work in milder conditions, and they are more environmentally friendly [4]. Among the enzymes, *Candida antarctica* lipase B (CalB) has shown a high catalytic activity for esterification of dicarboxylic acids [5, 6]. CalB is a versatile catalyst for a wide range of organic reactions [7]. Its high activity, thermostability, selectivity, and specificity compared to other known lipases make it special for unique applications [8].

In order to develop an efficient enzyme catalyzed process, knowledge of the substrate specificity is important [9]. The specificity of enzyme for different substrates cannot be predicted easily. Any factor that influences the enzyme-substrate binding or catalytic rate can affect the specificity of the enzyme [10]. So far, there are few studies on determining the substrate specificity of enzyme at the optimal value of all parameters influencing the reaction yield. Enzymatic synthesis of adipate esters using adipic acid and primary alcohols has been previously reported by [11, 12]. Furthermore, specificity of the enzyme for primary alcohols with different chain lengths, in the synthesis of adipate esters, has been previously studied [13]. In this study, specificity of the enzyme for alcohols of different classes, namely, primary, secondary, and tertiary alcohols, is investigated.

In the present study, response surface methodology (RSM) was used to optimize the reaction conditions. RSM is a useful statistical technique for optimizing multiple variables to predict best performance conditions using minimum cost and number of experiments [14]. It is used as a tool to assess the effects of several independent factors on the dependent variables. RSM has successfully been applied to study and optimize the enzymatic synthesis of various esters [15, 16].

In the present work, response surface methodology was used for studying the substrate specificity of immobilized *Candida antarctica* lipase B in esterification of adipic acid with different butanols. The effects of several reaction parameters on the synthesis of adipate esters were evaluated. The optimum conditions were obtained, and the effect of alcohol structure on the optimum conditions was investigated.

## 2. Materials and Methods

### 2.1. Materials

Novozym 435 (specific activity 10000 PLU/g; water content 1.4%) was purchased from NOVO Nordisk A/S (Bagsvaerd, Denmark) and consists of *Candida antarctica* Lipase B (triacylglycerol hydrolase, EC 3.1.1.3) immobilized on the macroporous acrylic resin (poly [methyl methacrylate-co-butyl methacrylate]. Adipic acid, iso-butanol (2-methylpropan-1-ol), sec-butanol, and tert-butanol (2-methyl-2-propanol) were purchased from Merck Co. (Darmstadt, Germany). n-Butanol was purchased from Sigma-Aldrich (St. Louis, MO, USA). All other chemicals and solvents used in this study were of analytical grade.

### 2.2. Lipase-Catalyzed Esterification

Different molar ratios of adipic acid and alcohol were mixed according to the experimental design, in 30 mL closed vials. Five milliliter of hexane was added as solvent [13]. Selection of hexane (log⁡ *P* = 3.5) as solvent was based on prior studies in which several solvents including hexane, heptane, acetone, ethyl acetate, butanol, and acetonitrile were screened for activity via lipase catalyzed esterification of adipic acid and different alcohols [17]. Different amounts of lipase, which were generated by RSM, were subsequently added. The reaction was performed in a temperature controlled (accuracy of ± 0.1°C) horizontal water bath at 150 rpm at different temperatures and for different time periods. The initial rates were calculated from the time profiles corresponding to the first minutes of the reaction (for which the profiles were approximately linear) and expressed as the amount of acid converted per unit of time per unit of weight of enzyme [18].

### 2.3. Analysis and Characterization

The reaction was terminated by dilution with 5 mL of ethanol: acetone (50 : 50 v/v), and lipase was removed by filtration. Remaining free acid in the reaction mixture was determined by titration with 0.1 M NaOH using phenolphthalein as the indicator. The moles of acid reacted were calculated from the values obtained for the control (without enzyme) and the test samples. The ester formed was expressed as equivalent to conversion of the acid [13]. Production of esters was characterized by FT-IR spectroscopy with absorption bands of C=O bend of ester at 1735, 1732, 1730, and 1690 cm^−1^ for di-n-butyl, di-iso-butyl, di-sec-butyl and di-tert-butyl adipate, respectively, and 1243, 1162, 1164 and 1189 cm^−1^ for C-O stretching vibrations of di-n-butyl, di-iso-butyl, di-sec-butyl, and di-tert-butyl adipate, respectively. Product was also monitored by gas chromatography/mass spectroscopy (GC/MS) on a Shimadzu (model GC 17A; model MS QP5050A; Shimadzu Corp, Tokyo, Japan) instrument with a BPX5 column (0.25 mm × 30 mm, 25 micron). According to GC/MS results, the reaction gave exclusive diester and formation of monoester was not observed. The mass spectrum of the products showed molecular ion at *m/z* 258 that corresponded to molecular formula C_14_H_26_O_4_. The two important ion peaks are related to the formation of ion asilium, [RCO]^+^, that gave the fragment ion at *m/z* 185 (because of the loss of alkoxy group from the ester, R-O) and the fragment ion at *m/z* 129 [O–CO–(CH_2_)_4_–C=OH]^+^ because of the rearrangement of the alkyl portion of the molecule. Other bonds cleavage occurred through some pathways and gave fragments ions at different *m/z*.

### 2.4. Experimental Design, Statistical Analysis, and Optimization

Response surface methodology (RSM) was applied to model the lipase catalyzed synthesis of adipate esters. To obtain a proper model for optimization, a four-factor-five-level central composite design (CCD) was employed, requiring 21 experiments. The fractional factorial design consisted of 8 factorial points, 8 axial points, and 5 center points. The variables and their levels selected for the adipate ester synthesis were temperature (35°C–65°C), time (30–420 min), amount of enzyme (20–400 mg), and substrate molar ratio (alcohol to adipic acid, 1 : 1–8 : 1). The design of experiments employed is presented in Table 1. Selection of the variables and their levels was based on the results obtained in our preliminary studies using one variable at a time approach. The experiments were produced in random order, and triplicate measurements of esterification yield were run on each experiment.

A software package of Design Expert Version 7.1.1 (State-Ease Inc., Statistics Made Easy, Minneapolis, MN, USA) was applied in this study. A second-order polynomial equation was developed to study the effects of the variables on the reaction yield

(1) $$y=b_{0}+\overset{4}{\underset{i=1}{\sum }}b_{i}x_{i}+\overset{4}{\underset{i=1}{\sum }}b_{ii}x_{i}^{2}+\overset{3}{\underset{i=j}{\sum }}\overset{4}{\underset{j=i+1}{\sum }}b_{ij}x_{i}x_{j}+e,\;$$

where *y* is the dependent variable (percentage of yield) to be modeled, *x*_*i*_ and *x*_*j*_ are the independent variables (factors), *b*_0_, *b*_*i*_, *b*_*ii*_, and *b*_*ij*_ are the regression coefficients of model and *e* is the error of model. The fit of the model was evaluated by coefficient of determination (*R* ^2^) and analysis of variance (ANOVA). The best-fitting model was determined by elimination of statistically insignificant terms until a significant model with an insignificant lack of fit was obtained.

## 3. Results and Discussion 

Fitting of the data to various models (linear, two factorial, quadratic, and cubic) and their subsequent ANOVA showed that synthesis of adipate esters were most suitably described with quadratic polynomial model. The quadratic polynomial models for the synthesis of different adipate esters are shown in Table 2. 

 The ANOVA for response surface models has been shown in Table 3. The very small *P*  value (<0.0001) and a suitable coefficient of determination (*R* ^2^ close to 1) show that the quadratic polynomial models are highly significant and sufficient to present the actual relationship between the response and the variables. According to ANOVA, the “lack of fit” is not significant at 95% confidence level indicating that the generated models are satisfactory, with acceptable predictive power [14]. 

The analysis of variance indicated that all the independent variables were statistically significant for the synthesis of di-n-butyl and di-iso-butyl adipate. The amount of enzyme did not have a significant influence on the synthesis of di-sec-butyl adipate. Temperature and substrate molar ratio were also not significant factors for the synthesis of di-tert-butyl adipate. However, these insignificant factors were not eliminated from the model equations. In case of di-sec-butyl adipate, eliminating the amount of enzyme resulted in a significant lack of fit. If a model has a significant lack of fit, it is not a good predictor of the response and should not be used [19]. In case of di-tert-butyl adipate, although temperature and substrate molar ratio were not significant, their dependents (quadratic effects of temperature and substrate molar ratio, temperature × substrate molar ratio, and enzyme amount × substrate molar ratio interactions) were significant and can affect the response. When an interaction is included in the model, its parent terms must also be included, even if they do not appear to be significant on their own, to maintain the model hierarchy [20]. 

**Figure 1:**
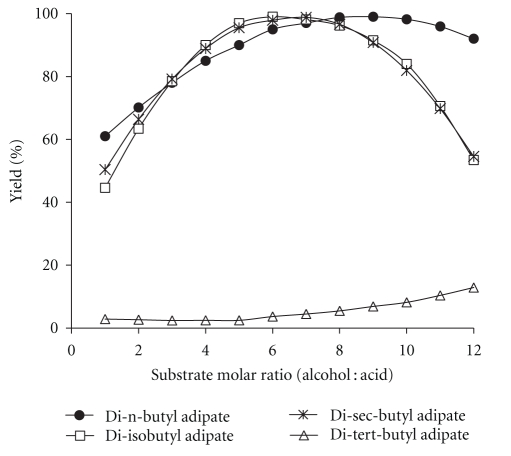
Effect of substrate molar ratio on the synthesis of adipate esters. Reaction conditions: temperature: 55°C, time: 250 min, and enzyme amount: 50 mg.

The equations shown in Table 2 were then used to study the effect of various parameters on the synthesis of adipate esters.  Figure 1 shows the effect of varying substrate molar ratio on the reaction yields. For all the alcohols, the percentage yield increased with increasing substrate molar ratio up to a certain amount. As alcohol concentration was raised, the ester yield continuously decreased except for di-tert-butyl adipate. The result is obviously a consequence of substrate inhibition that leads to decrease in the enzyme activity [21]. Alcohol inhibition of the lipase B from *Candida antarctica* has been previously reported [10]. According to Zaidi et al. [22], reaction between alcohol and enzyme leads to blocking of the nucleophilic site of the enzyme that is involved in the acylation process. The results indicate that no inhibition by tert-butanol can be seen up to alcohol: acid molar ratio 12 : 1. In fact, due to the significant steric hindrance by the methyl groups in the proximity of the hydroxyl group, the nucleophilic attack by tert-butanol becomes increasingly difficult. Therefore, the reactivity of alcohol and also its corresponding inhibition is very low. 

**Figure 2:**
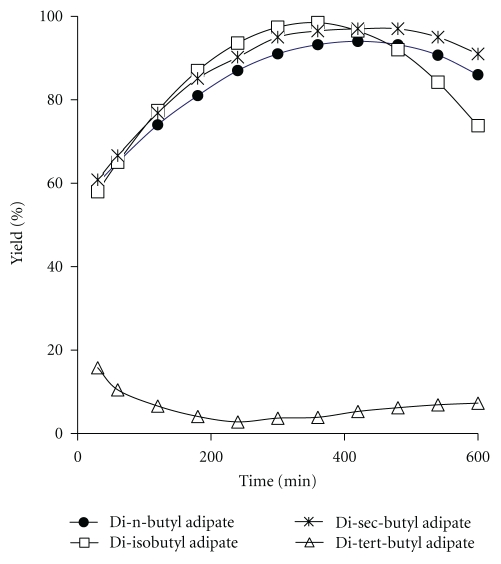
Time courses of adipate esters synthesis. Reaction conditions: temperature: 55°C, enzyme amount: 50 mg, and substrate molar ratio: 4.5 : 1.

Figure 2 represents the time courses of the enzymatic synthesis of adipate esters at 55°C, 50 mg enzyme and 4.5 : 1 substrate molar ratio. For all the esters except di-tert-butyl adipate, the percentage of yield increased with increase in incubation time up to an optimum amount. Prolonging the reaction time increases the volume of water produced by the reaction which leads to hydrolysis of ester [23]. Maximum production of di-tert-butyl adipate was observed within the first 30 minutes of the reaction. It can be concluded that time does not have a positive effect on the synthesis of di-tert-butyl adipate ester. This result also can be obtained from the predictive equation (Table 2) in which the factor of time has a negative coefficient. 

**Figure 3:**
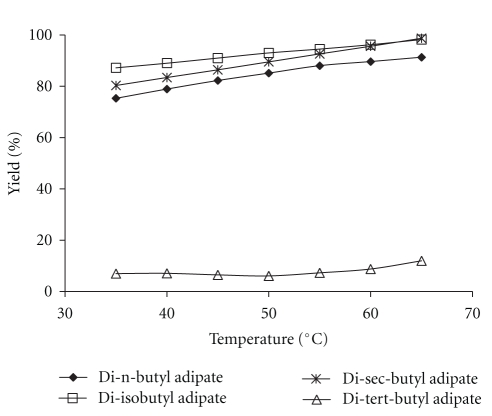
Effect of temperature on the synthesis of adipate esters. Reaction conditions: time: 250 min, enzyme amount: 50 mg, and substrate molar ratio: 4.5 : 1.

Figure 3 shows the effects of reaction temperature on esterification of adipic acid with different butanols at substrate molar ratio 4.5 : 1, amount of enzyme 50 mg, and time 250 min. The percentage of yields increased with increase in temperature within the given range (35°C –65°C). Higher temperatures increase the kinetic energy of the system and hence collisions between enzyme and substrate molecules to result in accelerated rates of the reaction [24]. An increase in temperature also improves solubility of the substrates and reduces viscosity, and mass transfer limitations resulted in enhancement of the reaction yield. Due to the evaporation of the solvent (boiling point of n-hexane = 68°C), higher temperatures were not considered for the reaction. Furthermore, high temperatures may also cause enzyme deactivation due to denaturation process. In the case of tert-butanol, by increasing the temperature, the reaction yield was only slightly increased, indicating that temperature is not a significant parameter in the synthesis of ester. This result is also confirmed by the ANOVA. 

**Figure 4:**
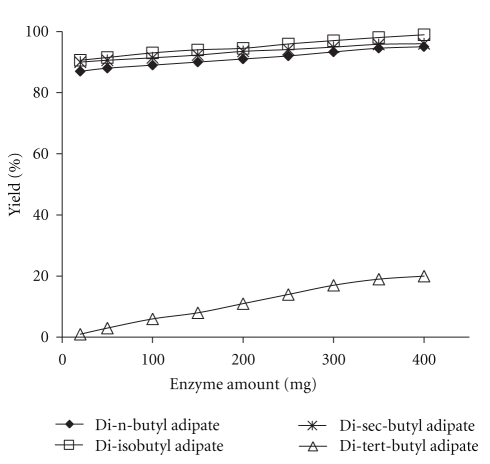
Effect of amount of enzyme on the synthesis of adipate esters. Reaction conditions: temperature: 55°C, time: 250 min, and substrate molar ratio: 4.5 : 1.

 The effect of varying amount of enzyme on the esterification reaction at 250 min, 55°C, and molar ratio of 4.5 : 1 is shown in Figure 4. An increase in enzyme concentration led to a marginal linear increase in esterification yield. Effect of enzyme amount seems to be more significant in the synthesis of di-tert-butyl adipate. According to ANOVA, *F*-value of the enzyme amount for the synthesis of di-tert-butyl adipate is 411.6, whereas for di-n-butyl, iso-butyl, and sec-butyl adipate, it is 14.4, 50.8, and 2.7, respectively. The presence of larger amounts of enzyme provides more active sites for acyl-enzyme complex formation [24]. 

From an economic point of view, the most efficient conditions for the reactions would be to use the lowest amount of enzyme to achieve the highest yield of ester. The predicted maximum percentage of yield using minimum amount of enzyme was 100.0% for di-n-butyl, di-iso-butyl, and di-sec-butyl adipate (Table 4). A yield of 42.8% was also predicted for the synthesis of di-tert-butyl adipate. Validation experiments carried out under predicated conditions showed good correspondence between experimental and predicted values. The obtained results imply that the maximum extent of esterification of adipic acid with primary and secondary alcohols is independent of the structure of alcohol. 

**Figure 5:**
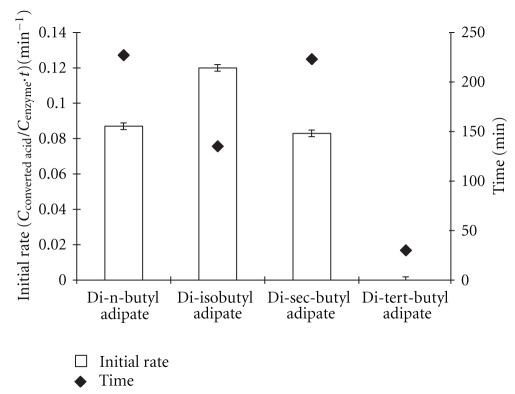
Effect of different classes of alcohols on the initial rate and minimum time required for maximum production of adipate esters. Reaction conditions: temperature: 62°C, enzyme amount: 35 mg, and substrate molar ratio: 7.7 : 1.

 Initial rates of the synthesis of adipate esters from different classes of alcohols are shown in Figure 5. Figure 5 also shows the required minimum time for maximum production of adipate esters at a reaction condition of 62°C, 35 mg enzyme amount, and substrate molar ratio 7.7 : 1. 

The highest initial rate (0.12 min^−1^) and shortest required time (135 min) were observed in the synthesis of di-iso-butyl adipate. This result is similar to the earlier findings by Deng et al. [25] that respecting the type of butanols in alcoholysis of alkyl esters, iso-butanol was superior to n-butanol as a substrate for CalB. However, it should be pointed out that this superiority is in the required time for synthesis of the esters and the extent of esterification is the same for both the alcohols. The initial rates of synthesis of di-n-butyl and di-sec-butyl adipate and also their required minimum time for maximum ester production (100.0%) were very similar. The result indicates that CalB has equal tendency for synthesis of these esters. The initial rate for the synthesis of tert-butyl adipate was nearly zero, and the maximum conversion yield (14.8%) obtained in 30 minutes was very low compared to other esters. According to Kourist and Bornscheuer [26], most of the commercially available enzymes do not accept tertiary alcohols as substrates. It was proposed that the configuration of the oxyanion hole in the lipase having bulky hydrophobic residues prevents activity towards tertiary alcohols with bulky structure.

## 4. Conclusion 

Optimization of several reaction parameters in Novozym 435-catalyzed esterification of adipic acid and different class of butanols was performed using response surface methodology. In this study, the specificity of the immobilized *Candida antarctica* lipase B for alcohols of different classes in the esterification of a short chain dicarboxylic acid was determined. A high percentage of yield, 100.0%, was predicted by the models for esterification of primary and secondary butanols implying that the maximum yield of esterification is independent of the alcohol structure for primary and secondary alcohols. Considering the substrate specificity behavior of the enzyme, structure of alcohol is an important parameter that can affect the optimized condition of other reaction parameters for the synthesis of adipate esters. The optimum conditions can also be used for future upscaling of the enzymatic production of adipate esters to obtain economically high-quality useful products at lower costs.

## Figures and Tables

**Table 1:** Range of variables for the central composite design.

Variable	Levels				
	−1.682	−1.000	0.000	+1.000	+1.682
Temperature, A (°C)	35.0	41.1	50.0	58.9	65.0
Reaction time, B (min)	30.0	109.0	225.0	340.9	420.0
Enzyme amount, C (mg)	20.0	97.0	210.0	323.0	400.0
Substrate molar ratio, D	1.0	2.4	4.5	6.6	8.0

**Table 2:** The predictive response equations relating yield of esterification to process parameters.

Ester	Model in terms of coded factors
Di-n-butyl adipate	*Y* = +86.56 + 5.10 *A* + 8.61 *B* + 2.60 *C* + 9.65 *D* − 2.96 *B* ^2^ − 2.62 *D* ^2^

Di-iso-butyl adipate	*Y* = +94.80 + 2.92 *A* + 10.66 *B* + 3.00 *C* + 12.30 *D* − 5.31 *B* ^2^−7.74 *D* ^2^ + 1.72 *AD* − 6.17 *BD* − 2.63 *CD*

Di-sec-butyl adipate	*Y* = +90.31 + 4.83 *A* + 9.96 *B* + 1.98 *C* + 12.93 *D* − 3.46 *B* ^2^−6.72 *D* ^2^ − 4.44 *BD*

Di-tert-butyl adipate	*Y* = +10.75 + 0.097*A* − 2.38 *B* + 5.48 *C* + 0.01 *D* + 4.00 *A* ^2^+2.36 *B* ^2^ + 0.78 *D* ^2^ − 2.21 *AD* − 1.16 *CD*

*Y *is the percent yield; *A* the temperature; *B* the time; *C* the amount of enzyme; *D* the substrate molar ratio.

**Table 3:** The analysis of variance (ANOVA).

Source	Sum of squares	Degree of freedom	Mean square	*F*-value	*P* value
*Di-n-butyl adipate*					
Model	2955.75	6	492.63	75.85	<0.0001
Lack of fit	61.65	10	6.17	0.84	0.6260
*R* ^2^ = 0.9702					

*Di-iso-butyl adipate*					
Model	5498.71	9	610.97	209.94	<0.0001
Lack of fit	29.12	7	4.16	5.75	0.0551
*R* ^2^ = 0.9942					

* Di-sec-butyl adipate*					
Model	5335.66	7	762.24	37.52	<0.0001
Lack of fit	244.27	9	27.14	5.47	0.0585
*R* ^2^ = 0.9528					

*Di-tert-butyl adipate*					
Model	764.28	9	84.92	48.26	<0.0001
Lack of fit	12.43	7	1.78	1.03	0.5221
*R* ^2^ = 0.9753					

**Table 4:** Optimum conditions for lipase-catalyzed synthesis of adipate esters.

Alcohol	Temperature	Time	Enzyme	Substrate	Predicted	Actual
	(°C)	(min)	amount (mg)	molar ratio	yield (%)	yield (%)
n-butanol	56	268	35	7.7	100.0	97.2
iso-butanol	54	220	20	6.0	100.0	97.0
sec-butanol	55	264	35	6.4	100.0	96.3
tert-butanol	65	30	350	3.8	42.8	39.1
